# Effectiveness of checklist-based box system intervention (CBBSI) versus routine care on improving postnatal care utilization in Northwest Ethiopia: a cluster randomized controlled trial

**DOI:** 10.1186/s12978-021-01283-9

**Published:** 2021-11-20

**Authors:** Netsanet Belete Andargie, Gurmesa Tura Debelew

**Affiliations:** 1 Ministry of Health, Addis Ababa, Ethiopia; 2 Department of Population and Family Health, Jimma University, Jimma, Ethiopia

**Keywords:** Box system, Postnatal care, Cluster randomized controlled trial, Ethiopia

## Abstract

**Background:**

The period after childbirth poses a substantial risk both to the mother and the newborn. Yet, this period received less attention as compared to the cares provided during pregnancy and childbirth. Hence, this study aimed to assess the effectiveness of checklist-based box system intervention on improving three postnatal care visit utilization.

**Methods:**

A double blind, parallel group, two-arm cluster randomized controlled trial design was used to assess effectiveness of checklist-based box system intervention on improving third postnatal care visit. Pregnant mothers below 16 weeks of gestation were recruited from 15 intervention and 15 control clusters, which were randomized using simple randomization. Data from baseline and end line surveys were collected using open data kit and analyzed using STATA version 15.0. The status of three postnatal care visit between intervention and control groups over time was assessed using difference in difference estimator. The predictors of the outcome variable were then analysed using mixed effects multilevel logistic regression model.

**Result:**

Of 1200 mothers considered from each of the baseline and end line studies, this study included data from 1162 and 1062 mothers at baseline and end line surveys, respectively. As it is shown from the difference-in-difference estimation (14.8%, 95%CI 5.4–24.2%, *p* = *0.002*) and the final model (AOR 4.45, 95%CI 2.31–8.54), checklist-based box system intervention was effective on improving third postnatal care visit. In addition, institutional delivery (AOR 1.62, 95%CI 1.15–2.28) and knowledge on danger signs during postnatal period (AOR 5.20, 95%CI 3.71–7.29) were found to be significant predictors of the outcome variable. In the contrary, mothers who got influenced by older generations of individuals were (AOR 0.32, 95%CI 0.18–0.59) less likely to attend three postnatal care visit.

**Conclusions:**

The implementation of checklist-based box system intervention was found to be effective in improving utilization of the recommended three postnatal care visits. The contribution of the trial on improving third postnatal care visit can be enhanced by minimizing practical level challenges, as well as expanding health messages to reach unreached mothers and significant others who can influence the mother’s decision.

*Trial registration*: ClinicalTrials.gov, NCT03891030, Retrospectively registered on 26 March, 2019, https://clinicaltrials.gov/ct2/show/NCT03891030.

## Plain language summary

Postnatal care is a care that a delivered women and her newborn baby receive after delivery and up to 42 days of childbirth, regardless of place of delivery. This period is considered critical in minimizing morbidity and mortality of both the mother and the baby. In Ethiopia, the coverage of postnatal care utilization remained low. Recent studies showed only 33.8% of mothers received postnatal care, this becomes very low for mothers who delivered at home, of which only 8.2% of them received the care. On top of other factors that hinder maternal health care utilization, in Ethiopia there are widely accepted and persistent cultural ceremonies after childbirth that encourages a women to stay in door.

This study introduced a new intervention called Checklist-based box system intervention, which aimed to improve postnatal care utilization through demand creation and dropout tracing mechanisms. The intervention was carried out by health professionals in health centers and community health workers (health extension workers) who visited mothers in their homes. Despite some practical challenges, its implementation demonstrated an improvement over clusters that did not receive the intervention. This intervention is recommended to be implemented on a larger scale. Simultaneously, practical level challenges need to be addressed in order for the intervention’s effect to be seen in its best form.

## Background

The world health organization (WHO) defines postnatal period as the period from the delivery of placenta and continues until 6 weeks or 42 days of delivery [1]. In this period major physical and psychological changes occur. Though the postnatal period is identified as a critical period for the lives of both the mother and newborns, it is the most neglected time when it comes to providing essential health services as compared to rates before and during delivery [2]. This limited service utilization by the mother and the newborn could result in ill health, disabilities and deaths [3].

In low and middle income countries, the use of postnatal care services remains highly inequitable [4]. In Africa, this period is marked by many cultural practices that keep mothers and newborns indoor [5]. Although postnatal care (PNC) has several benefits in reducing maternal and child morbidity and mortality significantly, its average utilization especially in sub-Saharan Africa (SSA) countries remained low (51.6%), similarly, in average 32.2% of women who did not deliver at a health facility had a postnatal check-ups [6]. In Ethiopia, only 8.2% of mothers who delivered at home received postnatal care [7].

Despite the progresses made in expanding the coverage of most of maternal health services, postnatal care coverage in Ethiopia is low, the result of three consecutive demographic and health surveys showed that the coverage of postnatal care is lower than institutional delivery and antenatal care four utilizations: (41.0%, 16.4%, 13.2%) [8], (32.0%, 26.0%, 17.0%) [9], (43%, 47.5%, 33.8%) [10] for antenatal care four, institutional delivery and postnatal care utilizations, respectively.

Literatures showed that previous maternal health visits, improved awareness and knowledge about the services given during maternal health visits were contributing for improved coverage of postnatal care visits [11–13]. In addition, it has been showed that, community engagement is essential to address any unsafe and irrational sociocultural beliefs and practices against PNC [14].

In Ethiopia, most studies have investigated the factors related to underutilization of postnatal care. However, there were very limited interventional studies, aimed to improve postnatal care utilization. So, identifying the greater importance of postnatal care for the lives of the mother and the newborn, checklist based box system interventions (CBBSI) were designed, implemented and tested with the aim of improving postnatal care utilization. The implementation of this study followed the Predisposing, Reinforcing, and Enabling Constructs in Educational Diagnosis and Evaluation (PRECEDE)–(Policy, Regulatory, and Organizational Constructs in Educational and Environmental Development) (PROCEED) framework as a lens. The framework underlined the importance of considering different approaches in the provision of culturally sensitive care [15]. Therefore, the aim of this study was to assess the effectiveness of checklist based box system interventions over the routine care on improving the recommended three postnatal care visits.

## Methods

The trial protocol for this study was published [16] and the trial was retrospectively registered on ClinicalTrials.gov, with trial identifier NCT03891030 on March 26, 2019.

### Design

A double-blind, parallel-group, two-arm cluster randomized controlled trial was conducted to assess the effectiveness of checklist based box system interventions on improving utilization of three postnatal care visits.

### Setting

This trial was conducted in Debre markos, Gozamin and Machakel districts of East Gojjam Zone, one of the administrative zones of Amhara region, located in North-western Ethiopia. In Ethiopia, women commonly receive maternal health services from primary health care units (PHCU), which are composed of a health center and satellite health posts. Health centers commonly staffed with Midwives and Nurses, while health posts are staffed with health extension workers. This PHCU linkage provides preventive and curative services to 25,000 people altogether [17–19]. By the time the trial begins health centers were staffed with a minimum of 2 midwives and more than 50% of health posts were staffed by two health extension workers.

### Sample size determination

The sample size calculation for this trial followed a recommendation for cluster randomized controlled trials with equal sized and fixed numbers of clusters [20]. Assumptions: three postnatal care visits in the control group was 16.0% [21], number of clusters available—30 (15 per arm), 95% confidence interval and 80% power, intra-cluster correlation coefficient of 0.04849 [22]. Then the sample size was calculated to determine number of observations per cluster using two sample comparisons of proportions using normal approximations and STATA 13 was used to run the calculation. Assuming individual randomization, the sample size per arm was 194, then allowing for cluster randomization, the average cluster size required was 40, and the final sample size was 1200 pregnant mothers (600 for intervention and 600 for control). Then 1200 mothers at the baseline and 1200 mothers during the end line survey were considered. The minimum detectable difference was calculated to be 12%.

### Randomization and sampling procedures

Debere-markos, Gozamin, and Machakel were chosen from among the sixteen districts available in Esat Gojjam Zone based on confirmation that none of the three districts had received an intervention/project aimed at improving maternal health service utilization. This trial has both community and facility level intervention. As the community level intervention would result information contamination, health posts/kebele under selected districts were used as a randomization units. After the inclusion of health posts/clusters, the study team used SPSS generated random sequence to assign the available 30 clusters to intervention and control arms in a 1:1 allocation ratio. In this study, identification and recruitment of health posts/clusters and identification of mothers for the baseline survey were done before the randomization procedure took place, which helps in minimizing identification bias.

### Participant eligibility criteria

Mothers who were pregnant and below 16 weeks of gestation, who were living in the selected districts were identified as participants for this trial. Mothers with severe clinical complications who required special ANC follow-up, on the other hand, were excluded from the study.

### Screening and enrolment

Mothers in the intervention group passed a two stage screening process to be enrolled in the study. First, community level pregnancy screening was done using Stanback et al. [23] pregnancy screening checklist, this community level screening identified suspected pregnant mothers. Then suspected pregnant mothers received a referral slip to be received by the nearby health center, for confirmation of pregnancy. Second, facility level screening took place at health centers using beta-human chorionic gonadotropin (HCG) urine test. Mothers who had confirmed pregnancy using the HCG test were enrolled in the study and received the first ANC on the same day. This community level survey, which was done by health extension workers (HEWs), was supported by a family folder: a documentation containing family profile of the residents at health post.

Unlike to the intervention group, health centers under the control group recruited self-referred pregnant mothers below 16 weeks of gestation and provide the first ANC on the same day.

### Blinding

Study participants (mothers) and outcome assessors were blinded to the intervention. Despite receiving all the listed packages of the intervention, mothers were unaware that they are in a different treatment group and receiving different intervention. The study team used the advantage of cluster randomization, where clusters rather than individuals are randomized. In this type of randomization, mothers who lived in the same cluster received the same intervention. Similarly, mothers who were under the intervention cluster of this study and were eligible for the service received CBBSI, which was common to all. In addition, health extension workers who were already familiar with the study's catchment population delivered the intervention.

Outcome assessors, data collectors, were blinded to the intervention. This study used a separate group of health care providers to provide the intervention and to collect the data. Health extension workers and health care providers who provided the intervention were involved in community level referral, health education sessions, managing referral slips, tracing dropouts, and providing a list of packages to be implemented in the intervention. This group of professionals was made aware of the intervention. However, a different group of health care workers meets mothers at their homes to collect data on their experiences during pregnancy, delivery, and the postnatal period for their index pregnancy. This group of health care providers who collected the data were unaware of, which group is receiving the intervention and which group is not. Furthermore, they are unaware of the reason for the intervention and the goals it is intended to achieve.

### Intervention description

#### Trial arm I/intervention

Checklist based box system intervention introduced a two pronged approach: community level demand creation and service utilization monitoring components. The trial was started with the work of HEWs, though community level identification of pregnant mothers. Mothers who had suspected pregnancy were linked to the nearby health center with a referral slip. On the same visit mothers were asked about their reasons of not attending maternal health services, and again to prioritize the top three reasons they had. These lists of reasons were documented using the reason picking card and placed at health posts’ health education scheduling box. Health extension workers provided individual health education based on local evidences: based on the mothers own reason for not utilizing maternal health services. Besides the person centered health educations, HEWs placed reason picking cards at health education scheduling boxes placed at health posts. These numbers of reason picking cards placed in each compartment determined the topic for health education that was delivered by HEWs in different platforms including pregnant mothers’ conferences.

At the receiving health center, mothers who have confirmed pregnancy received the first ANC care on the same day. Then mothers were expected to smoothly pass through the service utilization monitoring box placed at health centers: from ANC 1 to PNC 3. Mothers who fail to follow this smooth transition were identified by health care providers at health center and respective HEWs where the dropped mother belongs were communicated. Timing of postnatal visits followed the a recommendation from WHO and the nationally implemented three visit model: within 2 days of delivery (PNC 1), within 7 days of delivery (PNC 2) and within 42 days of delivery (PNC 3). The published protocol of this study includes a detailed description of the intervention [16].

#### Trial arm II/control

Mothers in the control arm received the existing routine care provided by the government. Accordingly, clusters in the trial arm II didn’t have community level pregnancy screening, health education scheduling box and person centered health education for health posts and service utilization monitoring box for health centers.

### Intervention process

The intervention followed previously developed intervention package, where each and every step of the trial with the necessary documentation was provided with. The intervention was supposed to be implemented in 10 months, but it took 20 months from the date the first mother with a confirmed pregnancy enrolled in the study and received the first ANC at the health center on January 3rd, 2019, to the end of follow-up for the last rounds of mothers on August 27th, 2020. This extended implementation period was following the practical level challenges on the ground, which are discussed further below. For a significant number of the cases, the family folder (used to guide community level pregnancy screenings) wasn’t updated, staff turnover and trained staff turnover at health posts and health centers was also a common problem. As it is clearly indicated in the protocol, for the purpose of follow-up of the identified suspected pregnant mothers, referral slip was filled in two copies and the first was given to the mother and the second was remained with the visiting HEW. Then these slips reached to health centers, where the mothers was referred using the weekly meeting platform between health posts and health centers. This weekly meeting went irregular and together with the above challenges the planned time for the trial was delayed.

### Data collection tools and techniques

The questionnaire used to collect data was designed incorporating different steps of the PRECEED-PROCED model as a base [15, 24]. The PRECEDE part focuses on the assessment of desired health outcome from different perspectives in four phases. Phase 1: Social assessment to identify desired outcome, Phase 2: Epidemiological assessment to set priority outcomes and assess determinants that stand in the way of achieving postnatal care three visit, Phase 3: Ecological assessments to identify predisposing, enabling and reinforcing factors that affect behaviour of a mother in receiving postnatal care and Phase 4: Administrative and policy assessment to identify administrative and policy issues that can influence the implementation of CBBSI. The PROCEED part also follows four phases: Phase 5: Design and implementation of CBBSI, Phase 6: Process evaluation to ensure the trial is implemented as planned, Phase 7: Impact evaluation to evaluate whether the intervention has contributed for the improvement of postnatal care utilization and Phase 8: evaluation to ensure if CBBSI is leading to the improvement in postnatal care utilization [25].

Then the tool was translated to the local language version of the study area. The study tool was pretested on mothers living outside of the chosen study area prior to data collection. Mothers who had their last child 1 year or less ago were chosen to take the pretest. A pretest assists the research team in re-examining and correcting the study tools (selected questions) for clarity, simplicity, and ordering. Then, the final tool was uploaded to kobo tool box and open data kit (ODK) was used to conduct face to face interview with selected mothers both for the baseline and end line study.

### Variables and measurement

The primary goal of this trial was to assess the effectiveness of a checklist-based box system intervention in increasing maternal health care utilization (ANC, skilled delivery and postnatal care). The first two primary outcomes had already been reported and were planned to be published. The findings of this study focused on reporting the effectiveness of the intervention on three postnatal care utilization. Accordingly, attending three postnatal care visits was the outcome variable for this study. Mothers were asked about the number of postnatal care visits or check-ups they had for their index delivery. In this study, Kebele was identified as a level variable and the independent variables were categorized as level 1 (individual level variables) and level 2 (community and facility level variables) variables (Table 1).

**Table 1:** Description of study variables, East Gojjam Zone, Northwest Ethiopia, January 2019–September 2020

Variables	Description	Measurement
Dependent variable		
Three postnatal visits/checkups	Attending three postnatal care visits to health facility/check-ups by health extension workers for the index delivery	Recoded as ‘Yes’ or ‘No’
Independent variables		
Level I variables		
Individual level variables		
Age	Age of the participant in completed years	A continuous variable and recoded into three categories 15–19, 20–29 and 30–49
Level of education	The highest level of education that the mother attended	Categorized in to four groups: non-formal education, primary education (1–8), secondary education (9–12) and above 12 grade
Marital status	Marital status of respondents	Categorized in to four categories: single, married, separated, widowed
Wealth quantile	Questions were adopted from EDHS and wealth index was computed using principal component analysis	Categorized in to five categories: poorest, poor, medium, rich and richest
Parity	Total number of deliveries a mother had	Categorized in to three categories: one, 2–4 deliveries and ≥ 5 deliveries
One postnatal care	Attending at least one postnatal care visits to health facility/check-ups by health extension workers for the index delivery	Coded as 'Yes' or 'No'
ANC four visit	Attending four and above consecutive health facility visit for pregnancy check-ups	Coded as 'Yes' or 'No'
Place of delivery	Participants were asked about the place they gave birth to their last baby	Coded as ‘Institution’ or 'Home'
Knowledge of danger signs of the postnatal period	Participants were asked to list danger signs of the postnatal period: severe headache, blurred vision, convulsions, swollen hands/face, high fever, loss of consciousness, difficulty breathing, sever weakness, foul smelling vaginal discharge	A composite index of these nine variables was created and dichotomized using the mean score: those who score the mean and above were categorized as *'Knowledgeable'*, those who score below the mean were classified as '*Not knowledgeable'*
Pregnancy wontedness	Participants were asked whether their last pregnancy was wonted or not	Coded as 'Yes' or 'No'
Compassionate and respectful care	A mother receiving MHC in a compassionate and respectful way: free of physical abuse, detention, non-confidential care, non-consented care, abandonment/neglect, and non-dignified care	Coded as 'Yes' or 'No'
Level 2 variables		
Community level		
Place of residence	The place where the respondent usually belongs	Coded as urban and rural
Average distance	Approximate distance of participants home from nearby health facility in minutes/on foot	A continuous variable, recoded in 2 categories: 0–30 min as '0' otherwise '1'
Influence of significant others in the process of receiving MHC	Participants were asked whether there is anyone who negatively influences them on the process of utilizing MHS at health facilities	Coded as 'Yes' or 'No'
Social support	Fourteen elements of SS questions: gets visits from significant others, getting useful advises, discussion on problems, having care at the time of labor and delivery, feeling loved, others thankful on them, getting help on household chores, help with money at emergency, help in transportation, card when sick, attending community level discussions, member of any religious cast, attending public meetings and help in case of conflicts	A principal component analysis was conducted, and a composite index was created using the principal components, and this was dichotomized using the mean score: those who score the mean and above were categorized as having *'Good social support'* and otherwise *'Poor social support'*
Facility level		
Receiving maternal health care free of charge	Respondents were asked about their facility MHC visit and the payment associated with it	Participants who always got the service free of charge coded as '1', most of the time as '2', and never '3'
CBBSI intervention	Kebele/clusters were identified as intervention and control based on the intervention (checklist based box system interventions on improving utilization of maternal health service utilization) received	Coded as intervention and control

### Data quality control

Before the actual field data collection training for a team of data collectors and supervisors took place, a manual explaining each and every question with its response categories and on how to work on ODK was prepared and provided for data collectors (BSC holder midwifes) and supervisors (MPH holder). In addition, the use of open data kit for data collection helped in settling questions as required (to avoid unanswered questions), in settling range checks for selected data values and allowed to deal with field editing before leaving the respondent.

### Data management and analysis

Data collection was managed centrally from Jimma University where, all study data bases were secured with password-protected access system. After the field data collection was over, data were exported from kobo-tool box and imported to STATA MP Version 15 for analysis.

Data from the trial followed an intention to treat analysis: participants were assigned to the cluster where they were resident when the trial begins. Allocation of clusters to the intervention and control arms followed simple randomization techniques, following this the treatment effect was analysed using the Difference in Difference (DiD) estimation [26]. Similarly, the status of utilization of three postnatal care visits between the intervention and control arms was tested using chi-square test for the significance of the association.

Then to identify factors affecting utilization of three postnatal care visits, first bivariate analysis was conducted to test the association between each independent variable and the outcome. Then, variables having p < 0.25 were included in the multivariable model.

The analysis employed multilevel mixed effect logistic regression; this model was selected because the participants of this study were nested in kebeles, which violates the assumption of independence of the ordinary logistic regression. Multilevel analysis allows the simultaneous examination of the effects of group level and individual level variables on individual level outcomes while accounting for the non-independence of observations within groups. Also, multilevel models took into consideration the dependency nature of individual probability in the areas of residence where the participants belong. This dependence on the context was accounted to obtain correct regression estimates.

First, an empty model was fitted to check for the presence of cluster level variability affecting the three postnatal care utilization and to measure the proportion of total variance that is attributable to cluster level, the intra-cluster correlation coefficient was calculated [27]. Additional measures of variation, median odds ratio (MOR) to measure unexplained cluster heterogeneity [27–31] and proportional change in variance (PCV) [32] were calculated. Models fitness for the multilevel was checked using the log likelihood ratio (LR) test.

Four models: an empty model: to evaluate the extent of cluster variation affecting three postnatal care utilization, second model: controlled for individual level variables, third model: controlled for community level variables and the fourth model controlled for both individual and community level variables were constructed. Variables with p value of < 0.05 in the second and third model were included in the final model. The p value of < 0.05 was used to define statistical significance, AOR together with 95% CI were used to show the strength of association and level of significance respectively.

## Results

### Profile of study participants

#### Baseline

**Fig. 1:**
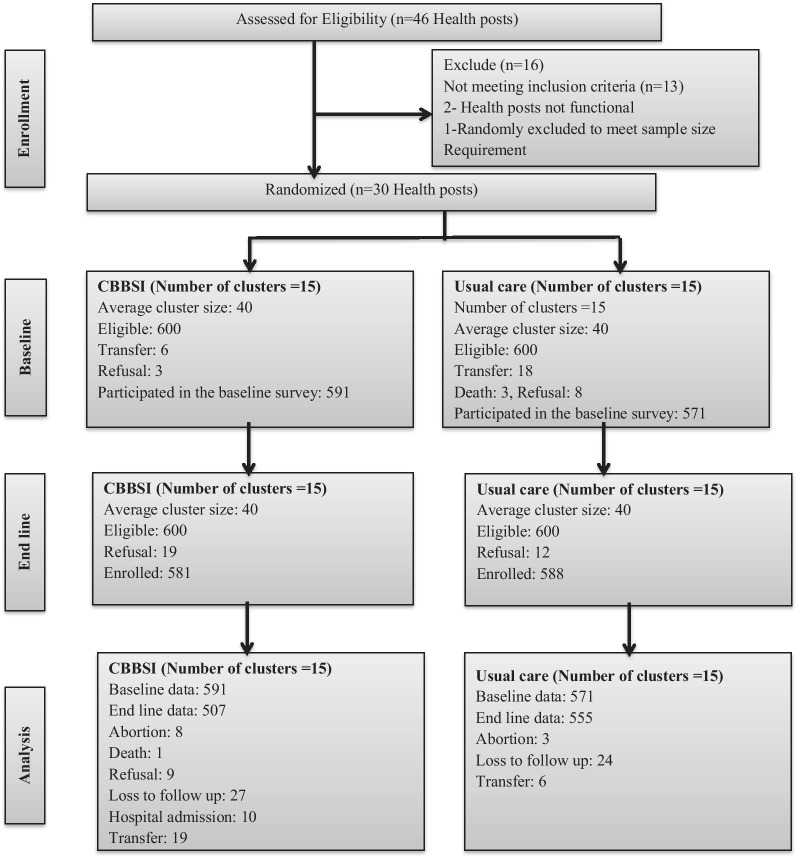
CONSORT flow diagram for cluster trials (this figure was used in another manuscript submitted to Trials with another objective)

Data from mothers belonging to 15 intervention and 15 control clusters were included in the analysis (Fig. 1). Baseline data were collected from 1162 participants, 591 (50.9%) from 15 control clusters and 571 (49.1%) from 15 intervention clusters. The mean age of study participants was 28.8 years (SD ± 6.1). Most participants were from rural residence 971 (83.6%) and in marital union 1108 (95.4%) (Tables 2, 3).

**Table 2:** Socio-demographic characteristics of study participants, East Gojjam Zone, Northwest Ethiopia, January 2019–September 2020 (n (baseline) = 1162, n (end line) = 1062)

Variable	Baseline (n = 1162)		End line (n = 1062)	
	Frequency	%	Frequency	%
Age				
15–19	50	4.3	39	3.7
20–29	591	50.9	697	65.6
30–49	521	44.8	326	30.7
Place of residence				
Rural	971	83.5	886	83.4
Urban	191	16.4	176	16.6
Educational status				
No formal education	331	28.5	284	26.7
Primary (1–8th grade)	724	62.3	623	58.7
Secondary (9–12th grade)	64	5.5	111	10.5
Above 12th grade	42	3.6	44	4.1
Marital status				
Single	30	2.6	16	1.5
Married	1108	95.4	1020	96.1
Separated	21	1.8	24	2.3
Widowed	3	0.3	2	0.2
Wealth Index				
Poorest	412	35.5	406	38.2
Poor	441	37.9	317	29.9
Medium	138	11.9	189	17.8
Rich	142	12.2	110	10.4
Richest	29	2.5	40	3.8
Parity				
One child	315	27.1	439	41.3
2–4 Children	620	53.4	519	48.9
≥ 5 Children	227	19.5	104	9.8

This table was included in a manuscript with another objective and submitted to Trials

**Table 3:** Baseline characteristics of intervention and control clusters Northwest Ethiopia, January 2019–September 2020 (n = 1162)

Variable	Intervention		Control	
	Frequency	%	Frequency	%
At least one PNC				
Yes	385	67.4	383	674.8
No	186	32.6	208	35.2
Three PNC				
Yes	232	40.6	221	37.4
No	153	26.8	162	27.4
Knowledge of danger signs of postnatal period				
Knowledgeable	178	31.2	208	35.2
Not-knowledgeable	393	68.8	383	64.8
ANC four attendance				
Yes	291	50.9	270	45.6
No	247	43.2	285	48.2
Place of delivery				
Health facility	268	46.9	260	43.9
Home	303	53.1	331	56.0
Level of social support				
Good	424	74.2	391	66.2
Poor	147	25.7	200	33.8
Influence by significant others				
Yes	42	7.4	7	1.18
No	529	92.6	584	98.8
Place of residence				
Urban	125	22.1	66	11.3
Rural	446	77.9	525	88.7
Pregnancy wontedness				
Yes	531	92.9	539	91.2
No	52	9.1	40	6.8
Compassionate and respectful care (n = 772)				
Yes	221	38.7	325	54.9
No	168	29.4	58	9.8

#### End line

During the end line survey data were collected from 1062 participants, 555 (52.3%) from 15 control and 507 (47.7%) from 15 intervention clusters. The mean age of study participants was 26.9 year (± 5.4) (26.7 and 27.3 years for control and intervention arms respectively). In the end line survey, most of the participants were also from rural residence 886 (83.4%) and in marital union 1020 (96.1%). Majority of the mothers 519 (48.9%) had 2–4 children (Table 2).

### Status of postnatal care utilization

#### Baseline

Most of the baseline study participants 772 (66.4%) had at least one postnatal care visit for their last baby. When participants were asked about the professionals attended their PNC visit, most 462 (59.8%) were seen by health care providers (Doctor, Health officer, Nurse, and Midwives), followed by health extension workers 271 (35.1%). Of the mothers who got PNC services, 385 (67.4%) from intervention and 383 (64.8%) from control clusters got one PNC visit, 298 (52.2%) from intervention and 299 (46.9%) from control clusters received two PNC visits and 232 (40.6%) from intervention 221 (37.4%) from control clusters got three PNC (Table 4).

**Table 4:** Comparison of PNC use between two arms of the baseline and end line respondents, Northwest Ethiopia, January 2019–September 2020 (baseline = 1162, end line = 1062)

PNC use	Baseline		End-line	
	Control clusters (n = 591)	Intervention clusters (n = 571)	Control clusters (n = 555)	Intervention clusters (n = 507)
No PNC visit	208 (35.2%)	186 (32.6%)	179 (32.3%)	68 (13.4%)
One PNC visit	383 (64.8%)	385 (67.4%)	376 (67.7%)	439 (86.6%)
Two PNC visits	299 (46.9%)	298 (52.2%)	277 (49.9%)	383 (75.5%)
Three PNC visits	221 (37.4%)	232 (40.6%)	205 (36.9%)	333 (65.6%)

The data included in the baseline assessment of this study refers to the data from another group of mothers who gave birth 1 year prior to the start of baseline data collection

#### End line

**Fig. 2:**
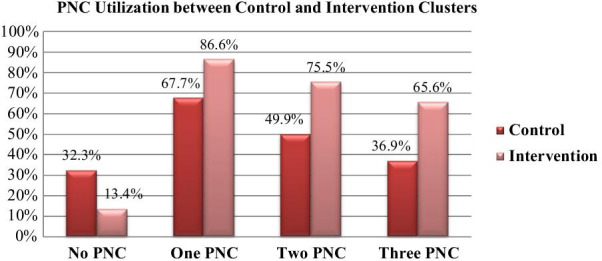
Status of utilization of postnatal care between the end line intervention and control clusters, East Gojjam Zone, Northwest Ethiopia

Among mothers participated in the end line survey, 815 (76.7%) had received at least one PNC, of whom 376 (67.7%) were from control and 439 (86.6%) were from intervention clusters. Regarding the professional who attended PNC visit 469 (57.5%) were seen by health care providers (Doctor, Health officer, Nurse, and Midwives), followed by health extension workers 154 (18.9%). Regarding the number of PNC visits 439 (86.6%) from intervention and 376 (67.7%) from control groups got one PNC visit, 383 (75.5%) from intervention and 277 (49.9%) from control clusters got two PNC visits and 333 (65.6%) from intervention and 205 (36.9%) from control groups received three PNC visits (Table 4, Fig. 2).

### Effectiveness of checklist based box system interventions on improving utilization of three postnatal care visits

The status of three postnatal care utilizations showed a significant improvement in the intervention clusters 333 (65.6%) as compared to controls 205 (36.9%), (*x*^2^ = 86.4, *p* < 0.0001) (Table 5). The result of the DiD estimation showed that, there is a statistically significant difference in the utilization of three postnatal care visits (14.8%, 95%CI 5.4–24.2%, *p* = *0.002*) between the intervention and control clusters over time.

**Table 5:** Postnatal care three utilization and related factors, Northwest Ethiopia, January 2019–September 2020 (n = 1062)

Variable	Intervention		Control		*x*^2^ test	*p* value
	Frequency	%	Frequency	%		
At least one PNC						
Yes	439	86.5	376	67.7	52.69	< 0.0001
No	68	13.4	179	32.3		
Three PNC						
Yes	333	65.6	205	36.9	86.38	< 0.0001
No	174	34.4	350	63.1		
Knowledge of danger signs of postnatal period						
Knowledgeable	276	54.4	276	49.7	2.35	0.13
Not-knowledgeable	231	45.6	279	50.3		
ANC four attendance						
Yes	432	85.2	287	51.7	135.9	< 0.0001
No	75	14.8	268	48.3		
Place of delivery						
Health facility	403	79.5	323	58.2	55.5	< 0.0001
Home	104	20.5	232	41.8		
Level of social support						
Good	344	67.9	404	72.8	3.11	0.07
Poor	163	32.1	151	27.2		
Influence by significant others						
Yes	60	11.8	36	6.5	9.22	< 0.01
No	447	88.2	519	93.5		
Place of residence						
Urban	131	25.8	45	8.1	60.24	< 0.0001
Rural	376	74.2	510	91.9		
Pregnancy wontedness						
Yes	475	94.2	523	93.7	0.14	0.71
No	32	5.8	32	6.3		
Compassionate and respectful care (n = 953)						
Yes	450	90.7	433	94.7	5.7	0.02
No	46	9.3	24	5.3		

### Factors affecting utilization of three postnatal care visits

To evaluate the applicability of the model, first the empty model of the mixed effect multilevel logistic regression analysis was run and it showed that of the total variation in the utilization of three postnatal visits across clusters/kebele, 22.6% (ICC: 22.6%: *p* value < 0.0001) of it was attributed to cluster level. Similarly, MOR of 2.69 [2.69 ≠ 1 (no difference)] showed the presence of area level variation in the utilization of three postnatal care visit, indicating the data set is best fit for the mixed effects multilevel logistic regression.

In model 2, after adjustment for individual level (Level 1) factors, the variance attributable to cluster level was slightly reduced to 20.9% (ICC:20.9%: *p* value < 0.0001, MOR:2.43). Similarly, in model 3 after controlling for community level (Level 2) factors, the variance attributable to cluster level was slightly reduced to reach to 19.3% (ICC:19.3%: *p* < 0.01), MOR:2.23). Finally, in model four, after adjusting for both individual and community level factors, the area level variance reached to 16.8% (ICC:16.8%: *p* < 0.05, MOR:2.01) (Table 6). With regard to the explained variation, 30.3% of the variation was explained by the full model.

**Table 6:** Random intercept model/measure of variation for postnatal care three utilization, Northwest Ethiopia, January 2019–September 2020

Measure of variation	Model 1	*p* value	Model 2	*p* value	Model 3	*p* value	Model 4	*p* value
Variance (SE)	0.96 (0.39)	< 0.0001	0.87 (0.29)	< 0.0001	0.79 (0.27)	< 0.0001	0.66 (0.22)	< 0.0001
ICC (%)	22.6		20.9		19.3		16.8	
MOR	2.69		2.43		2.23		2.01	
PCV	*Reference*		9.1		18.2		30.3	
Model fitness statistics								
Deviance	− 660							
LR test vs logistic model (p-value)	< 0.0001							

Model 1: intercept only model. Model 2: adjusted for individual level variables. Model 3: adjusted for community level variables. Model 4: adjusted for individual and community level variables

*SE* standard error, *ICC* intra cluster correlation coefficient, *MOR* median odds ratio, *PCV* explained variation

In the final model, among the included variables, two from level two (cluster label) (receiving CBBSI intervention and influence of significant others) and two variables from level one (individual label), (place of delivery and knowledge about danger signs of pregnancy) were significantly associated with the outcome variable (Table 6).

Mothers who were under the intervention clusters and received CBBSI (AOR 4.45, 95%CI 2.31–8.54) were more likely to attend three postnatal visits as compared to those who were under the control arm and received the usual care. Mothers who delivered at institutions (AOR 1.62, 95%CI 1.15–2.28) were more likely to attend three postnatal visit/check-up as compared to those who delivered at home. Similarly, mothers who were knowledgeable about danger signs of the postnatal period (AOR 5.20, 95%CI 3.71–7.29) were more likely received three postnatal visit/check-up.

Mothers who were influenced in the process of receiving maternal health care (AOR 0.32, 95%CI 0.18–0.59), on the other hand, were less likely to receive three postnatal care visits (Table 7).

**Table 7:** A multilevel logistic regression analysis of factors affecting fourth antenatal care utilization, Northwest Ethiopia, January 2019–September 2020 (n = 1062)

Variables	Model 2 AOR (95%CI)	Model 3 AOR (95%CI)	Model 4 AOR (95%CI)
*Individual level factors*			
Age			
15–19	1		
20–29	2.46 (0.97–6.24)		
30–49	1.99 (0.74–5.37)		
Educational status			
Non-formal	1		
Primary (1–8th grade)	0.91 (0.59–1.40)		
Secondary (9–12th grade)	1.04 (0.57–1.92)		
Above 12	0.47 (0.18–1.23)		
ANC four attendance			
No	1		
Yes	1.41 (0.99–1.99)		
Place of delivery			
Home	1		1
Health facility	**1.51 (1.04–2.19)**		1.62 (1.15–2.28)**
Knowledge of danger signs of postnatal period			
Not-knowledgeable	1		1
Knowledgeable	**4.62 (3.18–6.69)**		5.20 (3.71–7.29)***
Compassionate and respectful care			
No	1		
Yes	1.54 (0.81–2.92)		
Pregnancy wontedness			
No	1		
Yes	0.79 (0.38–1.66)		
*Community level factors*			
Place of residence			
Rural		1	
Urban		0.77 (0.39–1.55)	
Influence by significant others			
No		1	1
Yes		**0.15 (0.08–0.27)**	0.32 (0.18–0.59)***
Level of social support			
Poor		1	1
Good		**2.53 (1.79–3.56)**	1.29 (0.88–1.89)
CBBSI cluster			
Control		1	1
Intervention		**5.22 (2.61–10.42)**	4.45 (2.31–8.54)***

Estimates in bold refer to significant values

Model 1: intercept only model, **p < 0.01, ***p < 0.0001

## Discussion

The implementation of checklist based box system intervention was found effective on improving utilization of three postnatal care visits. Mothers who received CBBSI were more likely to receive three postnatal visits compared to their counterparts. This study used the PRECEDE–PROCEED model as lens which indicates: variations on personal desires might need individualized care. Accordingly, individual health education sessions coupled with service utilization monitoring approaches of this trial contributed for the improved utilization of three postnatal care visits, as compared to the usual care, where mass health educations are being done without dropout tracing mechanisms. To make the implementation of this trial smooth: proper documentation, clear and functioning PHCU linkage, continuous monitoring and tracing of dropouts need to be ensured. Similarly, once suspected mothers were identified and linked to health centers, availability of the confirmatory test (HCG) need to be secured.

The result of this study showed that mother’s place of delivery had contributed for attending three PNC visits, this finding goes in line with a comparative report from developing countries that showed low proportion of mothers who delivered at home went for postnatal visits [6, 33]. In Ethiopia, mothers are encouraged to deliver at health center and hospitals, where they can get skilled care at the intrapartum and postpartum periods. For mothers who deliver at home for different reasons, a family member is expected to report to the nearby health extension workers for home level postnatal check-up. HEWs were also expected to visit mothers who delivered at home for PNC starting from the first visits and to provide consecutive postnatal check-ups for mothers who deliver at health facilities. Literatures showed that the number of mothers who visited health facilities for maternal health services decreased as a mother goes from ANC to facility delivery and to the postnatal period [34]. Mothers who delivered at health facilities were those who were likely attended ANC services and those who had PNC visits were those who were likely delivered at health institutions. As the number of maternal health care visits is increasing, the contact between a mother and health care providers also increased which in turn increases health seeking behavior of a mother gives time to create clear understanding of what was said by health care providers.

According to the finding of this study, mothers who were classified as knowledgeable about danger signs in of the postnatal period were more likely to attend postnatal visit. This finding goes in line with a study conducted in Benchi-Maji zone [13], which reported awareness of the problems of the postnatal period as a predictor of postnatal care service utilization. This finding indicated, programs aiming to improve maternal health care utilization need to relook the way mothers are being reached for health education and counseling during antenatal care visits or need to design targeted interventions to increase women’s awareness about the importance of maternal health care visits in general and postnatal care visits in particular.

This study showed that mothers who got influenced in the process of receiving maternal health services by close relatives like mother, grandmother and mother-in-law were less likely to complete the recommended postnatal visits/checkups. Similar studies also showed the negative influence of significant others in the process of receiving maternal health services [35]. Mostly close relatives had a significant influence on the mother; this population group at the same time was not the focus of programs which are aimed to improve maternal health services utilizations. On top of this, like other developing countries [5], postnatal care utilization in Ethiopia is greatly affected by a culture that encourage mothers to stay indoor after delivery. Different cultural practices are held immediately after delivery and continued for consecutive days and weeks. These practices were greatly affecting the rate of utilization of postnatal care visits. Until a concreate and regular behavioral change communications will reach to mothers and significant others who can influence their decision, the home visit made by health extension workers can be seen as an option for those who didn’t visit health facilities for PNC. However, the quality of care provided by HEWs should be relooked again.

In an empty model of the mixed effect multilevel logistic regression analysis, of the total variation 22.6% (ICC = 22.6%, *p* < *0.0001*) of it is attributable to cluster level, and MOR of 2.69 also confirmed that utilization of three postnatal care visits is attributable to cluster level variations. However, the ICC and MOR in model 2 and 3 adjusted for individual and community level factors respectively and model 4 adjusted for both individual and community level factors showed a little drop in ICC and MOR as compared to the null model. Similarly the proportional change in variance showed that, only 30% of the variations were explained by the full model. These values indicate that there were also unexplained variations between communities that are contributing for the utilization/non-utilization of the three postnatal care visits (Table 6).

## Strength and limitations

One of the strength of this study is that, it employed a double-blind approach. Mothers and outcome assessors were blinded to the intervention. Mothers were unaware that they are in a different treatment group and receiving different intervention. Furthermore, the study used different groups’ health care providers to deliver the intervention and to collect data. The data collectors were blinded to the intervention, which groups belonged to the intervention and control groups, and the hypothesis that would be tested. In this process the study limits the occurrence of ascertainment bias or detection bias. The randomization procedure of this study used simple randomization techniques. Taking this type of randomization into consideration, the analysis employed a difference in difference estimation to estimate the difference in three postnatal care utilization among the intervention and control group over time, baseline and end line periods. In addition, before the randomization procedure, health posts/clusters were identified and recruited, and mothers were identified for the baseline survey. This helped to reduce identification bias.

However, there is may be a probability of self-reporting or recall bias as a limitation. The study team used study tools that had been pretested with a similar group of mothers for clarity and understandability. To assess the outcome variable, mothers were asked the following question, ‘How many postnatal check-ups did you have for your most recent delivery?' which is thought to be clear, understandable, and not leading. Despite the above-mentioned considerations, as long as there is self-reporting, there might be a possibility of self-reporting bias. Furthermore, the study used a comparable study period of 1 year to meet mothers who participated in the baseline and end line studies. This cut off was chosen with the natural course of pregnancy, delivery, and the postnatal period in mind. Despite the fact that the time interval between delivery and study participation would have similar effects for both baseline and end line studies, it could still be associated with recall biases.

## Implications for public health practice

The implementation of CBBSI has introduced an intervention that can be scaled up both in urban and rural settings. The demand creation component of the intervention, which was delivered at individual bases, is believed to increase knowledge of mothers, which in turn increases postnatal visits. Also service utilization monitoring component of the intervention has contributed in tracing mothers who fail to abide with the recommended visits. To ensure smooth screening and enrollment, the CBBSI implementation must accompany and plan for the challenges encountered on the documentation system, PHCU linkage, trained professional turnover, and continuous availability of supplies and equipment at health post and health center level.

The finding of this study showed that knowledge is one of the significant predictor for three postnatal care visits. In an area where majority of women of reproductive ages didn’t access at least one of the media outlets commonly used by the government to transmit health messages [9], health care providers and health extension workers counseling especially during pregnancy need to be strengthened. We suggest the adequate and continuous delivery of health messages during such platforms. In addition the implementation of checklist based box system intervention introduced individual health education sessions for pregnant and delivered mothers and mother in their postnatal period, as there is a culture of staying at home after delivery; this is especially recommended for mothers in their postnatal period and believed to improve both for maternal and neonatal outcome.

Health education sessions are recommended to be extended to significant others (husband, mother, grandmother and mother in laws) who have an influence on the decision of a mother to go for postnatal visits and check-ups. This approach, in addition to increasing the utilization rate it also supports the community level linking of home delivered mothers for PNC check-ups by HEWs.

On top of delivering postnatal care by HEWs and working to increase knowledge of the mother and her extended families, contextualized and culture specific interventions need to be considered. In addition, the work of HEWs needs to be supported by trainings, use of equipment and quality of care components while delivering home level postnatal care check-ups. Finally, the final model of this study showed there are still factors that are contributing for the utilization of three postnatal care visits that remain unexplained, which need to be investigated further.

## Conclusion

The implementation of checklist based box system intervention showed a significant improvement on the utilization of three postnatal care visits over the usual care. The implementation of this trial can be boosted through minimizing practical level challenges: improving documentation at health post level, uninterrupted supply of necessary materials, improving the knowledge and skill of health extension workers and strengthening the linkage between primary health care units. On top of designing context and culture specific interventions to improve utilization of postnatal care, population groups who potentially could influence the decision of the mother need to be reached.

## Data Availability

The datasets used and/or analysed during the current study are available from the corresponding author on reasonable request.

## Abbreviations

ANC: Antenatal care
CBBSI: Checklist based box system interventions
DiD: Difference in difference
EDHS: Ethiopian demographic and health survey
HCG: Human chorionic gonadotropin
HEW: Health extension worker
ICC: Intra cluster correlation coefficient
MDD: Minimum detectable difference
MOR: Median odds ratio
ODK: Open data kit
PCV: Proportional change in variance
PRECEDE: Predisposing, Reinforcing, and Enabling Constructs in Educational Diagnosis and Evaluation
PROCEED: Policy, Regulatory, and Organizational Constructs in Educational and Environmental Development
SSA: sub-Saharan Africa
WHO: World Health Organization
